# Interpretable Machine Learning for Emergency Department Triage: Clinical Insights from 133,198 Patients Using the Korean Triage and Acuity Scale (KTAS)

**DOI:** 10.3390/diagnostics16060954

**Published:** 2026-03-23

**Authors:** MyoungJe Song, Jongsun Kim, Eun-Chul Jang, SoonChan Kwon

**Affiliations:** 1 Department of Emergency Medicine, International St. Mary’s Hospital, Catholic Kwandong University, Incheon 22711, Republic of Korea; smj66@ish.ac.kr; 2 Department of Occupational and Environmental Medicine, Soonchunhyang University Cheonan Hospital, Cheonan 31151, Republic of Korea; oemdr10@gmail.com (E.-C.J.); 91ksc@hanmail.net (S.K.)

**Keywords:** emergency department triage, Korean triage and acuity scale (KTAS), decision-making, machine learning, explainable artificial intelligence (XAI)

## Abstract

**Background/Objectives:** Emergency room severity classification (KTAS) is essential for patient safety but has limitations due to its reliance on subjective judgment. Existing machine learning models have not been trusted in clinical settings due to their opaque ‘black box’ nature in decision-making processes. To overcome this, this study aims to develop an explainable machine learning framework that provides a transparent basis for judgment with high accuracy. **Method:** We retrospectively analyzed 133,198 emergency room visits from 2022 to 2024. We trained Random Forest (RF) and XGBoost models using vital signs and pain scores and applied explainable AI (XAI) techniques to ensure model transparency. **Results:** Although XGBoost showed the highest predictive performance (94.7% accuracy within a ±1 error margin), we ultimately selected the RF model, which provides a good balance of predictive power (91.6%) and interpretability for clinical use. The results of the XAI analysis confirmed that pain score, age, and systolic blood pressure were the key variables in prediction, strongly aligning with clinical logic. **Conclusions:** This study demonstrates that explainable AI can provide transparent insights for KTAS prediction beyond the limitations of traditional black-box models. These models may support emergency department triage by improving consistency and assisting clinicians in identifying potentially high-risk patients. However, further external validation is required before routine clinical implementation.

## 1. Introduction

Initial triage in the Emergency Department (ED) is a pivotal gateway that determines the trajectory of patient care. It sets treatment priorities and allocates scarce medical resources based on the severity of the patient’s condition. This process is fundamental to maintaining both patient safety and the operational efficiency of the healthcare system [1,2,3]. As patient volumes continue to rise globally, the pressure on EDs has intensified, making accurate and rapid triage more critical than ever.

However, current triage systems are not without significant flaws. Traditional frameworks often rely heavily on the subjective judgment, intuition, and clinical experience of the triage nurse. While valuable, this reliance introduces variability; factors such as cognitive fatigue, high-stress environments, and varying levels of experience can lead to inconsistencies. Such subjectivity can result in under-triage (classifying a critical patient as stable), which endangers patient safety, or over-triage (classifying a minor issue as critical), which strains resources and contributes to ED overcrowding [4]. These issues are particularly exacerbated during patient surges, pandemics, or when patient history is incomplete.

To mitigate these human-centric limitations, research into Artificial Intelligence (AI) and Machine Learning (ML) has seen exponential growth [5,6,7]. Various standardized triage frameworks, such as the Canadian Triage and Acuity Scale (CTAS), the U.S. Emergency Severity Index (ESI), and the U.K. Manchester Triage System (MTS), have been digitized and analyzed. Despite their structured nature, concerns persist regarding their inter-rater reliability and predictive accuracy in real-world, high-volume settings [8,9].

Consequently, ML models trained on Electronic Medical Records (EMR) and initial vital signs have emerged as a promising alternative. Ensemble learning methods, specifically Random Forest (RF), Gradient Boosting Decision Tree (GBDT), and Extreme Gradient Boosting (XGBoost, version 1.7.6), have demonstrated superior predictive capabilities compared to traditional regression models [10,11]. For instance, Jiang et al. [12] highlighted that XGBoost significantly outperformed conventional logistic regression in predicting admission and mortality.

However, a high-performing model is insufficient if clinicians cannot trust it. The “black-box” nature of complex algorithms has shifted the research focus toward eXplainable AI (XAI). Interpretability is non-negotiable in clinical settings where decisions can be life-or-death. Techniques such as permutation importance, SHapley Additive exPlanations (SHAP), and Partial Dependence Plots (PDPs) are increasingly being integrated into triage research to bridge the gap between algorithmic accuracy and clinical trust [13,14,15,16].

The Korean Triage and Acuity Scale (KTAS) is the standard five-level tool used across Korean emergency departments. Despite this widespread use, studies leveraging large-scale datasets to automate or validate KTAS using interpretable artificial intelligence (AI) remain scarce [17,18]. To explicitly address the limitations of subjective triage and the opaque nature of existing machine learning models, the primary objective of this study is to develop a highly accurate and trustworthy machine learning framework for KTAS prediction using a massive dataset of 133,198 ED visits recorded between 2022 and 2024. The fundamental novelty of this work lies in directly bridging the gap between algorithmic performance and clinical applicability by employing Explainable AI (XAI) techniques. Furthermore, by rigorously evaluating the model’s clinical utility and interpretability beyond mere prediction, we propose a practical and transparent decision-support tool for frontline clinicians.

## 2. Materials and Methods

### 2.1. Study Design and Data

This study employed a retrospective observational design. The dataset was extracted from the Electronic Medical Records (EMR) of a tertiary university hospital in South Korea, covering the period from January 2022 to December 2024. Data from all consecutive patients who visited the emergency department during that period were initially collected to clarify the data acquisition process. Subsequently, in order to ensure the ground truth of the predictive model and increase the reproducibility of the research workflow, we strictly excluded DOA patients or cases with missing initial KTAS labels. Consequently, a total of 133,198 visits were included in the final analysis. To reflect the real-world operational flow of an ED, each visit was treated as an independent case, regardless of whether it was a repeat visit by the same patient. The study protocol underwent rigorous review and was approved by the Institutional Review Board (IRB). Given the retrospective nature of the study and the use of fully de-identified data, the requirement for informed consent was waived.

### 2.2. Variable Definition and Preprocessing

The input features were carefully selected based on data available at the exact moment of triage. These indicators were chosen because they are information that can be collected immediately and non-invasively upon arrival in the ER before blood tests or imaging diagnostic results are available. This is to reflect the actual time constraints of the initial severity classification process in the model. These included:

- Demographics: Gender and Age.
- Vital Signs: Systolic Blood Pressure (SBP), Diastolic Blood Pressure (DBP), Heart Rate (HR), Respiratory Rate (RR), and Body Temperature (BT).
- Symptom Assessment: Pain score measured via the Numerical Rating Scale (NRS).

To capture physiological interplay, two derived indicators were calculated:

- Mean Arterial Pressure (MAP): Calculated as MAP = (SBP + 2 × DBP)/3, offering a better indicator of perfusion to vital organs than SBP alone.
- Shock Index (SI): Calculated as SI = HR/SBP, a sensitive marker for occult shock and hemodynamic instability.

Data preprocessing was conducted in a stepwise manner as follows. First, we cleaned physiologically impossible outliers (e.g., HR > 300 or <0). Second, minor missing values were imputed using median replacement to maintain dataset integrity. Median imputation was explicitly chosen for handling missing values because it is more statistically robust than mean imputation against clinical outliers commonly occurring in emergency department environments [19,20].

### 2.3. Label Definition

The ground truth label for the model was the initial KTAS level assigned by the certified triage nurse or emergency physician upon patient arrival. The classification adhered strictly to the guidelines of the Korean Society of Emergency Medicine. The five levels are defined as follows:

- Level 1 (Resuscitation): Cardiac arrest, major trauma, respiratory arrest (Immediate intervention).
- Level 2 (Emergent): Potential threat to life or limb (Care within 10 min).
- Level 3 (Urgent): Condition may progress seriously if untreated (Care within 30 min).
- Level 4 (Less Urgent): Patient requires intervention but is stable (Care within 1–2 h).
- Level 5 (Non-Urgent): Minor ailments requiring no immediate intervention (Care within 2 h).

For the purpose of binary classification performance analysis, we also grouped Levels 1–2 as “High Acuity” (Critical) and Levels 3–5 as “Non-High Acuity” (Stable). This binary classification scheme was introduced to validate the model’s ability to distinguish between patients in relatively stable conditions and those requiring immediate life-saving interventions. This aims to reflect the practical clinical priorities of high-volume emergency settings.

### 2.4. Modeling Strategy

We selected Random Forest (RF) as the primary model for this study due to its effective balance between performance and interpretability. This is an essential component of clinical decision-making. RF is advantageous for capturing complex, nonlinear interactions between vital signs, effectively reflecting the characteristics of data that traditional linear models may miss. RF operates by constructing a multitude of decision trees and outputting the mode of the classes. This ensemble approach effectively reduces overfitting. For comparative validation, XGBoost (version 1.7.6) was also deployed. XGBoost is a gradient-boosting framework known for its execution speed and model performance, particularly on large structured data sets, providing a robust benchmark for our primary RF model.

Regarding the data partition, we utilized a 70/30 split for training (70%) and testing (30%) sets. This specific ratio was chosen because it is a standard practice widely accepted in medical machine learning research. This is to ensure a sufficient amount of data for robust learning of the model, while also ensuring a large, independent test set that allows for rigorous evaluation of generalization performance in environments similar to real-world clinical settings [20].

The model optimization and validation followed a systematic, step-by-step process. First, hyperparameters—such as tree depth, learning rate for XGBoost, and number of estimators for RF—were optimized using a Grid Search method. Second, this optimization was integrated with 5-fold cross-validation. This was done to minimize bias in performance due to specific data partitioning and to ensure the model maintains stable predictive power. This rigorous approach ensures that the final models are robust and generalize well to unseen clinical data.

### 2.5. Performance Evaluation Metrics

To provide a holistic view of model performance, four distinct metrics were utilized. This multi-faceted evaluation approach was adopted to comprehensively verify the statistical accuracy of the model, as well as its practicality in real-world emergency clinical settings and its impact on patient safety.

- Quadratic Weighted Kappa (QWK): This was the primary metric, as it penalizes discrepancies between predicted and actual levels. Unlike standard accuracy, QWK accounts for the ordinal nature of triage scales (e.g., mistaking Level 1 for Level 5 is worse than mistaking Level 1 for Level 2). Since KTAS levels represent ordinal data, it is essential to reflect the clinical risk associated with the distance between grades. Therefore, QWK was selected to statistically quantify the severity of misclassification.
- Mean Absolute Error (MAE): Computed as the average absolute distance between the predicted class and the true class. MAE intuitively demonstrates how many levels the model’s predictions deviate from the actual severity rating on average, which is useful for quantifying the magnitude of the overall prediction error.
- ±1 Accuracy: This clinical metric reflects the percentage of predictions that fell within one level of the true label, acknowledging that triage often has a “grey zone” of subjectivity. This was included to evaluate the practical utility of the model, taking into account acceptable variations in clinical judgment that may occur in real-world practice.
- Confusion Matrix Analysis: Used to visualize specific patterns of error, specifically identifying rates of under-triage (dangerous) versus over-triage (inefficient). By analyzing the direction of misclassification beyond simple numbers, we aimed to verify the safety of the model by precisely examining the prevalence of under-triage that threatens patient safety and over-triage that hinders ED operational efficiency.

### 2.6. Interpretability Analysis (XAI)

To demystify the “black box,” we applied three interpretability techniques. These multi-faceted XAI approaches were adopted to validate that the model’s predictive rationale aligns with real-world medical knowledge and clinical intuition, thereby providing a basis for medical staff to trust the model’s decisions.

- Permutation Importance: We systematically shuffled the values of each feature to measure the resulting drop in QWK. This identifies which variables are most heavily relied upon by the model. This technique measures the actual contribution of each variable to predictive performance without requiring model re-training, making it effective for determining overall variable importance.
- Partial Dependence Plots (PDPs): These plots visualize the marginal effect of a specific feature (e.g., Age) on the predicted outcome, holding other variables constant. This helps verify if the model follows biological logic (e.g., does risk increase as age increases?). PDPs were used for the post hoc validation of whether the nonlinear relationships learned by the model conformed to physiological logic. For instance, we confirmed the clinical feasibility of the model by checking whether the predicted severity risk increased as vital sign parameters deteriorated.
- Uniform Manifold Approximation and Projection (UMAP): A dimension reduction technique used to project the high-dimensional patient data into a 2D scatter plot. This allows us to visually compare the clustering of the model’s predictions against the actual patient distribution. UMAP is highly efficient for visualizing high-dimensional clinical data because it preserves both the local and global structures of the data. This facilitated an intuitive assessment of how effectively the model differentiates between various KTAS categories.

## 3. Results

### 3.1. Characteristics of the Study Population

The final analysis included 133,198 unique ED visits. The demographic profile showed a median age of 43 years (Interquartile Range [IQR]: 21–63), with a slight female predominance (52.2%). The distribution of acuity levels was skewed, as is typical in general ED populations:

- High Acuity (Levels 1–2): 13.2% (Level 1: 2.8%, Level 2: 10.4%)
- Mid Acuity (Level 3): 52.2% (The dominant category)
- Low Acuity (Levels 4–5): 34.7% (Level 4: 26.9%, Level 5: 7.8%)

Detailed baseline characteristics, including vital sign averages per group, are tabulated in Table 1. As the acuity level increased (from KTAS 5 to KTAS 1), there was a noticeable trend of advancing age and more unstable physiological parameters, particularly in heart rate and shock index.

### 3.2. Model Performance Comparison

Both ensemble models demonstrated robust predictive capabilities, particularly in the “near-miss” metric of ±1 accuracy, where both exceeded 90%.

- XGBoost Results: Demonstrated the highest raw statistical power with a QWK of 0.476, MAE of 0.386, and Exact Accuracy of 67.4%. Its ±1 Accuracy was 94.7%, indicating it rarely made catastrophic errors.
- Random Forest Results: While slightly lower in raw metrics (QWK = 0.434, MAE = 0.494, Accuracy = 61.0%, ±1 Accuracy = 91.6%), RF was selected as the primary model.

Rationale for Selection: Despite the statistical superiority of XGBoost, RF was chosen because it prioritized clinical accountability. The decision-tree structure of RF is much easier for clinicians to intuitively understand, and its method of calculating variable importance remains more stable. In real emergency clinical settings, the ability to provide a reliable basis for predictive outcomes is as essential as the performance of the model itself.

**Figure 1:**
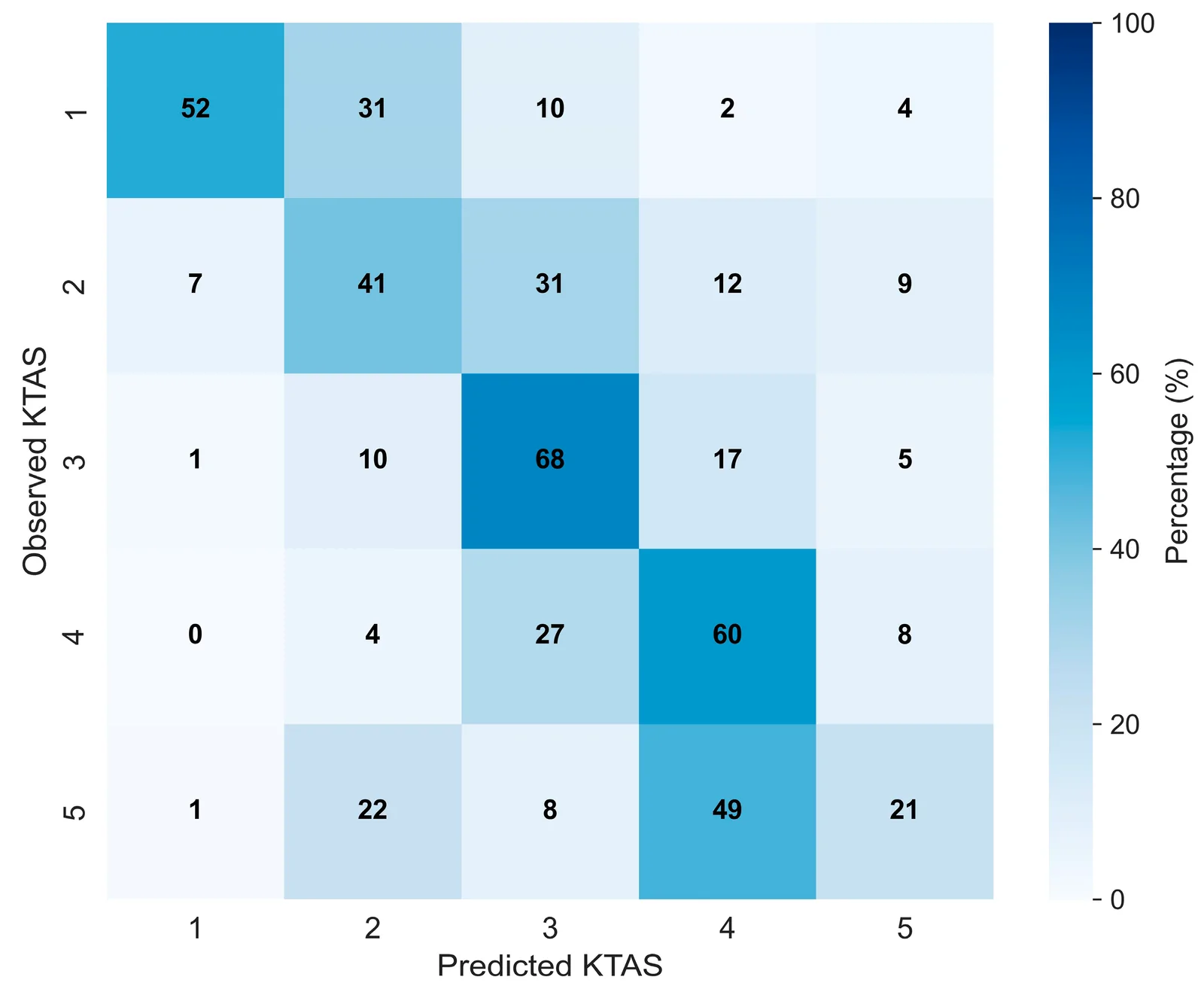
Confusion matrix of Random Forest predictions for the Korean Triage and Acuity Scale (KTAS). The heatmap displays the row-normalized classification percentages. The method was adopted to precisely evaluate classification performance within each class without bias, taking into account the classification imbalance specific to the ER dataset. The *x*-axis represents the predicted KTAS levels, and the *y*-axis represents the observed (actual) KTAS levels. The diagonal elements indicate the recall (sensitivity) for each class. Darker blue indicates a higher percentage of agreement.

Confusion Matrix Analysis (Figure 1): The RF model excelled at classifying the majority class (Level 3). However, distinct error patterns emerged:

- Under-triage (21.7%): Cases where the model predicted a lower severity than the nurse. This is the primary safety concern.
- Over-triage (17.4%): Cases where the model predicted higher severity.
- Exact Match (61.0%): Perfect agreement. Crucially, the majority of misclassifications occurred between adjacent severity levels (e.g., Level 2 vs. Level 3), and fatal errors such as misclassifying Level 1 as Level 5 were extremely rare. This suggests that the model shows substantial agreement with expert triage decisions when evaluated using the ±1 tolerance metric (>90%).

### 3.3. Variable Importance and Clinical Plausibility (Figure 2)

The permutation importance analysis revealed that the model does not rely on noise but on clinically significant markers. These results show that the predictive rationale of the model is consistent with emergency medical priorities, demonstrating a strong link between the technical nature of the data and its real-world clinical significance.

- Pain Score (NRS): The single most predictive feature. This means that the model has accurately learned real-world clinical guidelines where pain is utilized as a key indicator of severity determination in the KTAS classification system.
- Age: Highly significant, reflecting the higher biological vulnerability of older adults.
- Systolic Blood Pressure (SBP): A key indicator of hemodynamic status.

Partial Dependence Plots (PDPs) Analysis: The PDPs confirmed that the model learned non-linear, medically accurate relationships:

- Pain: The probability of High Acuity rose sharply as NRS increased.
- Age: Risk increased progressively with age, with a steeper incline after age 60.
- SBP: A “U-shaped” non-linear relationship was observed. Specifically, the probability of high-acuity classification increased during both hypotension (suggesting potential shock) and severe hypertension (suggesting hypertensive emergencies).

These nonlinear patterns demonstrate that the ensemble model successfully captures medical complexities that are difficult to identify using simple linear regression models. This alignment with established clinical knowledge further validates the model’s reliability for real-world emergency department deployment.

**Figure 2:**
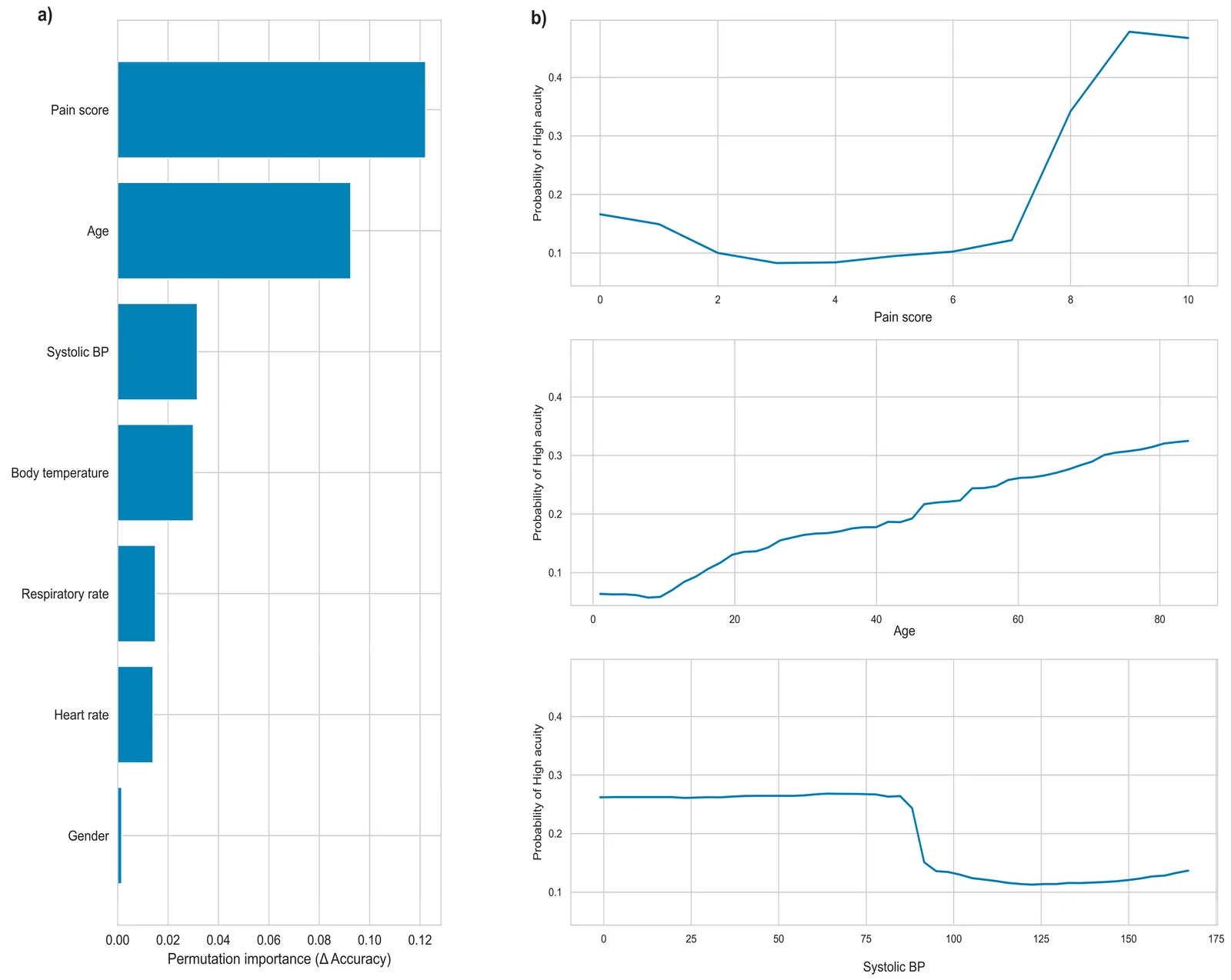
Explainability analysis of the Random Forest model. This was done to resolve the black-box properties of artificial intelligence and to verify that the model’s predictive rationale matches medical knowledge and clinical intuition to ensure the model’s reliability. (**a**) Permutation importance ranking of input variables. Pain score and Age were identified as the most significant predictors. (**b**) Partial Dependence Plots (PDP) illustrating the marginal effect of key features on the predicted probability of high acuity (KTAS Level 1–2). The plots show non-linear relationships: risk increases with higher pain scores (**top**), advances with age (**middle**), and rises significantly at low systolic blood pressure levels (**bottom**). The pattern of a sharp rise in risk, especially in the hypotension section, demonstrates that the model has accurately learned the medical principle of hemodynamic instability.

### 3.4. Patient Distribution Visualization (UMAP)

**Figure 3:**
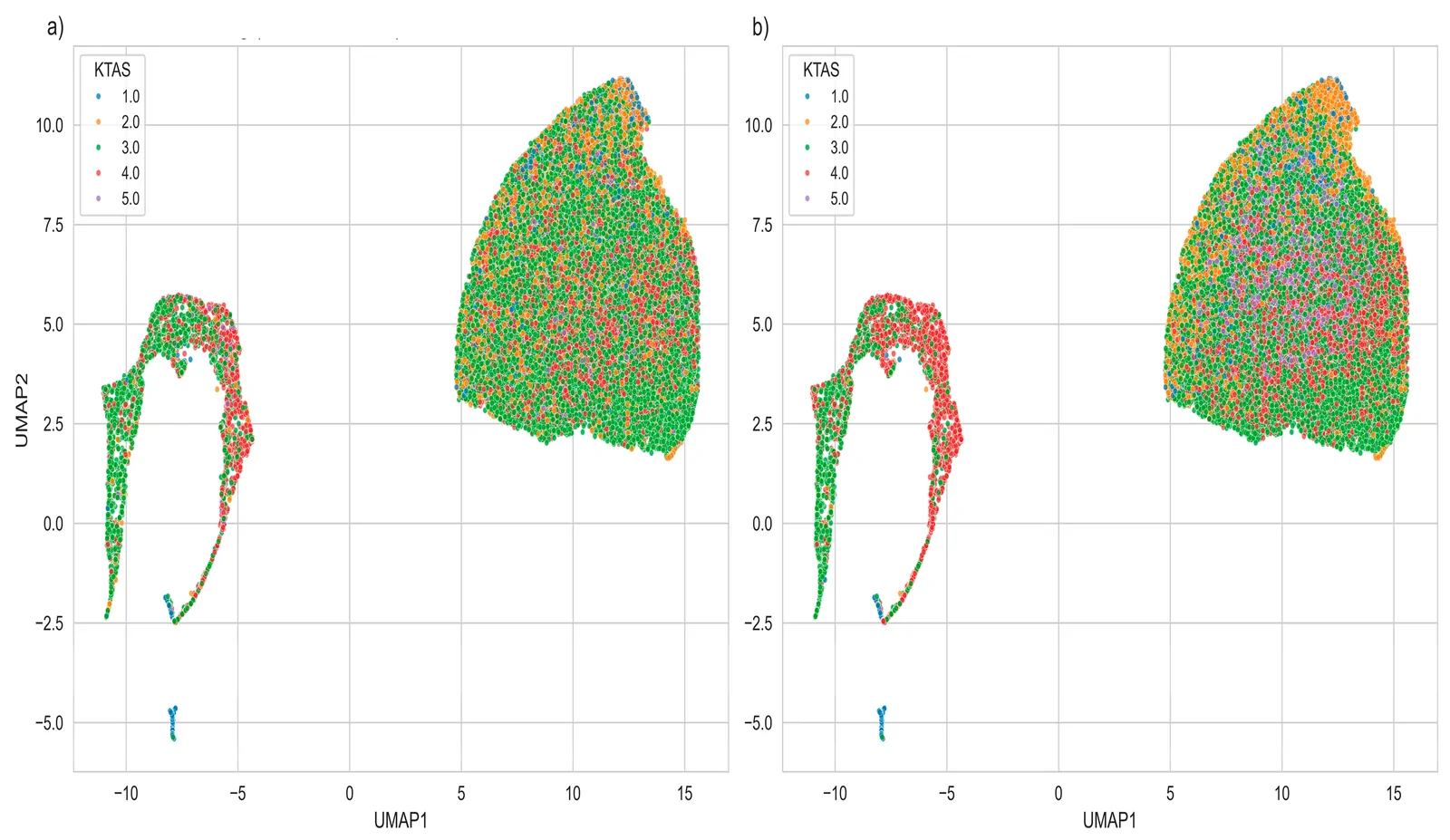
UMAP Visualization of Patient Distribution. Dimensionality reduction plots comparing (**a**) Actual KTAS labels and (**b**) Predicted KTAS labels. This analysis was used to visually confirm that the global topology inherent in high-dimensional clinical data is maintained without distortion in the prediction results of the model. Each dot represents a patient visit projected into 2D space. The visual structure of the predicted labels closely mirrors the actual distribution, confirming that the model learned the underlying data topology. However, the significant overlap between adjacent levels (e.g., Level 2 and 3) in the actual data explains the misclassification rates observed in the confusion matrix. This overlapping phenomenon suggests that patient severity exists on a continuous spectrum rather than a discontinuous grade in real emergency medical sites, which supports the clinical feasibility of model misclassification patterns.

The UMAP (Figure 3) was utilized to visualize the 133,198 visits in a low-dimensional space to verify the model’s global understanding of the patient population. This visualization was used to examine whether meaningful patterns and separations between KTAS levels were preserved in the high-dimensional clinical data.

The Actual Label map showed distinct, though overlapping, clusters for each KTAS level. The Predicted Label map mirrored this topography closely, confirming that the model captured the underlying structure of the data. However, the observed overlap suggests that vital signs and demographics alone may not be sufficient to perfectly separate complex Level 2 and Level 3 patients. This overlapping phenomenon suggests that patient severity in a real-world emergency setting exists on a continuous spectrum rather than discrete categories, reflecting the inherent complexity of clinical judgment. This indicates a need for incorporating additional features, such as chief complaint text, in future modeling iterations.

## 4. Discussion

### 4.1. The Need for Objective Triage Support

Cardiovascular and cerebrovascular diseases remain the leading causes of mortality worldwide [1,2,3], and timely identification in the ED is the single biggest determinant of survival. However, the traditional “human-only” approach to triage using systems like ESI, KTAS, and MTS has shown vulnerabilities. Studies have repeatedly demonstrated that triage nurses, while highly skilled, are susceptible to fatigue, cognitive bias, and environmental stressors, leading to variability in care [4,8,21]. Recent studies show that ER overcrowding further intensifies this variability, adding to the cognitive load of triage nurses, significantly increasing misclassification rates, especially in high-severity situations [22]. Multi-center investigations confirm that inter-rater reliability for these manual systems is often only moderate (Kappa 0.4–0.6) [23,24,25]. Several previous studies have explored machine learning approaches for emergency department triage prediction [26,27,28]. Many of these studies primarily focused on predicting downstream outcomes such as hospital admission or mortality rather than replicating triage decisions themselves [27,28]. In contrast, our study focuses on interpretable machine learning for KTAS prediction using a large real-world dataset, with an emphasis on model transparency and clinical interpretability.

### 4.2. Interpreting the Model Performance

In this analysis of more than 130,000 cases, ML models demonstrated reasonable agreement with expert triage decisions, particularly when considering the ±1 tolerance metric (>90%). However, exact agreement remained moderate (61%), and the clinical impact of under-triage requires careful consideration. While XGBoost provided a slight edge in raw accuracy (QWK 0.476), the Random Forest model offered a comparable performance with superior interpretability characteristics. The selection of Quadratic Weighted Kappa (QWK) as the main endpoint in this study is a very valid analytical decision in the clinical context. This is because, unlike simple accuracy, QWK strictly reflects the ordinal nature of medical severity data and imposes a strong penalty on misclassification between distant classes (e.g., determining Level 1 as Level 5), which can have a devastating impact on actual patient safety [29]. The identification of Pain Score, Age, and SBP as top predictors aligns perfectly with clinical intuition—pain acts as a universal alarm, age correlates with frailty, and blood pressure dictates perfusion [30].

However, the 21.7% under-triage rate warrants careful consideration. In a clinical setting, missing a Level 2 patient (under-triage) is far more dangerous than upgrading a Level 4 patient (over-triage). This suggests that while the model is accurate, the decision threshold in the algorithm might need to be tuned to favor “sensitivity” (catching all sick patients) over “specificity” for real-world safety. Nevertheless, the overall grade agreement and accuracy within tolerance (±1) shown by this model are equal to or partially above the average inter-rater reliability of skilled human nurses reported in recent emergency triage evaluation studies [31].

### 4.3. The Role of Explainable AI (XAI) in Adoption

The most significant barrier to AI adoption in healthcare is trust [32]. A “black box” model that outputs “Level 1” without explanation will likely be ignored by a busy physician. The development of XAI is the strategic solution to this [33,34]. Global health organizations, including the World Health Organization (WHO), have specified transparency and explainability as key ethical principles in the adoption of AI in healthcare [35]. We have transformed our model from a simple black-box calculator to a clinical consultant who presents logic transparently by integrating permutation importance and partial dependence plots (PDPs) that efficiently provide global and local interpretability without the undue computational burden required by complex frameworks.

### 4.4. Clinical Implications and Future Directions

Our findings suggest that ML-based triage should function as a “Digital Second Opinion” rather than a replacement for nurses. As emphasized by Rajpurkar et al. [34] and Greenhalgh et al. [36], the success of AI depends on its integration into the hospital’s social and organizational fabric, a perspective aligned with Sendak’s stepwise implementation framework [37]. To advance this field, future work must extend beyond single-center studies to national validation using multi-center datasets, ensuring the model is free from demographic bias [38]. Ensuring algorithmic fairness in healthcare AI requires preventing operational biases confined to specific healthcare institutions from becoming entrenched, which essentially requires robust external validation covering various demographic characteristics and healthcare systems [39]. Furthermore, integrating unstructured data—specifically “Chief Complaint” text via Natural Language Processing (NLP)—is crucial for capturing clinical nuances missed by vital signs alone. Recent advances in Large Language Model (LLM) and Natural Language Processing (NLP) technologies demonstrate that the integration of patient verbatims into analytical models can dramatically improve triage accuracy by effectively capturing the nature of subjective pain and complex clinical contexts [26]. Ultimately, research should evolve from simply replicating triage levels to predicting hard outcomes, such as hospital admission and mortality, to assess whether AI can surpass human standards in risk identification [27,28].

## 5. Limitations

Several limitations of this study should be acknowledged. First, as a single-center study, the data reflect the demographic characteristics and disease prevalence of a specific region, limiting the generalizability of our findings. Second, the model relied exclusively on structured data (vital signs and demographic information). The exclusion of chief complaint text from the analysis likely constrained the model’s ability to detect emergencies with subtle clinical nuances (e.g., a stroke patient presenting with normal vitals but slurred speech). Third, given the retrospective nature of this research, evaluating real-time interactions between the model and clinical staff was not feasible. Finally, this study utilized the nurse’s triage score as the ground truth. Consequently, if the initial assessment was inaccurate, the model inevitably learned to replicate those errors. Future investigations should incorporate actual patient outcomes (e.g., mortality or ICU admission) as the reference standard.

## 6. Conclusions

This study successfully developed and validated an interpretable machine learning framework for KTAS prediction using a large-scale dataset. We demonstrated that while XGBoost offers marginal gains in accuracy, Random Forest provides a pragmatic balance of performance and explainability essential for clinical trust. By visualizing the “why” behind predictions through XAI, these models may provide supportive insights for emergency department triage, potentially reducing variability and assisting clinicians in identifying high-risk patients and allocating emergency resources.

## Figures and Tables

**Table 1:** Baseline characteristics by Korean Triage and Acuity Scale (KTAS) level.

Variable	Overall (*n* = 133,198)	KTAS 1 (*n* = 3672)	KTAS 2 (*n* = 13,803)	KTAS 3 (*n* = 69,476)	KTAS 4 (*n* = 35,802)	KTAS 5 (*n* = 10,445)
Age, years	43.0 (21.0–63.0)	73.0 (59.0–83.0)	62.0 (45.0–76.0)	42.0 (21.0–62.0)	30.0 (8.0–53.0)	48.0 (34.0–61.0)
Gender, *n* (%)						
Male	63,707 (47.8)	1983 (54.0)	7548 (54.7)	31,292 (46.5)	18,042 (50.4)	3847 (37.0)
Female	69,491 (52.2)	1689 (46.0)	6255 (45.3)	37,184 (53.5)	17,760 (49.6)	6598 (63.0)
Systolic BP, mmHg	122 (103–139)	99 (55–134)	123 (99–146)	124 (106–142)	119 (93–136)	123 (116–131)
Diastolic BP, mmHg	75 (60–86)	55 (24–77)	73 (57–86)	76 (62–87)	74 (44–85)	78 (71–84)
MAP, mmHg	91.3 (75.7–103.0)	71.0 (32.0–96.0)	90.3 (72.0–105.0)	92.0 (78.0–104.3)	89.3 (64.3–101.7)	93.3 (87.3–98.3)
Heart rate, bpm	89 (75–107)	86 (52–110)	87 (73–105)	91 (76–110)	90 (77–107)	76 (70–86)
Respiratory rate,/min	18 (16–20)	20 (16–23)	18 (16–20)	18 (16–20)	18 (16–20)	16 (16–18)
Body temperature, °C	36.8 (36.4–37.3)	36.4 (35.0–37.1)	36.7 (36.3–37.1)	36.9 (36.5–37.6)	36.8 (36.4–37.1)	36.7 (36.4–36.9)
Shock Index	0.6 (0.5–0.8)	0.8 (0.6–1.0)	0.7 (0.5–0.9)	0.6 (0.5–0.8)	0.6 (0.4–0.7)	0.6 (0.5–0.7)
Pain score (NRS)	2.0 (0.0–4.0)	0.0 (0.0–0.0)	0.0 (0.0–4.0)	4.0 (0.0–5.0)	2.0 (0.0–4.0)	0.0 (0.0–0.0)

Values are presented as median (interquartile range) or *n* (%). BP, Blood pressure; MAP, Mean arterial pressure; NRS, Numerical Rating Scale.

## Data Availability

The dataset analyzed in this study is derived from the electronic medical records (EMR) of the Catholic Kwandong University International St. Mary’s Hospital. Due to institutional and legal restrictions protecting patient privacy, the data cannot be shared publicly. De-identified data may be made available upon reasonable request from the corresponding author, subject to institutional review and IRB approval.
